# Face Adaptation Effects: Reviewing the Impact of Adapting Information, Time, and Transfer

**DOI:** 10.3389/fpsyg.2013.00318

**Published:** 2013-06-03

**Authors:** Tilo Strobach, Claus-Christian Carbon

**Affiliations:** ^1^Department of Psychology, Humboldt-University, Berlin, Germany; ^2^Department of Psychology, Ludwig-Maximilians-University, Munich, Germany; ^3^Department of General Psychology and Methodology, University of Bamberg, Bamberg, Germany

**Keywords:** face adaptation, figural adaptation effects, memory representation, learning, plasticity, perception, transfer, delay

## Abstract

The ability to adapt is essential to live and survive in an ever-changing environment such as the human ecosystem. Here we review the literature on adaptation effects of face stimuli to give an overview of existing findings in this area, highlight gaps in its research literature, initiate new directions in face adaptation research, and help to design future adaptation studies. Furthermore, this review should lead to better understanding of the processing characteristics as well as the mental representations of face-relevant information. The review systematizes studies at a behavioral level in respect of a framework which includes three dimensions representing the major characteristics of studies in this field of research. These dimensions comprise (1) the specificity of adapting face information, e.g., identity, gender, or age aspects of the material to be adapted to (2) aspects of timing (e.g., the sustainability of adaptation effects) and (3) transfer relations between face images presented during adaptation and adaptation tests (e.g., images of the same or different identities). The review concludes with options for how to combine findings across different dimensions to demonstrate the relevance of our framework for future studies.

The term *adaptation* refers to the ability to adjust to novel information and experiences. This ability to adapt is essential for living and surviving in an ever-changing environment such as the human ecosystem (Carbon and Ditye, 2012). Visual adaptation in particular is an effect of the processes by which the visual system encodes and represents information, and includes a process by which the visual system (passively or actively) alters its function in response to the lack of fit between mental representations and perceived objects (e.g., Clifford et al., 2000; Clifford and Rhodes, 2005). Such responses result in adaptation effects. In experimental situations, such adaptation effects are typically assessed in an adaptation test phase after an adaptation phase. Intensive investigations of these adaptation effects provides an excellent opportunity for an exploration and deeper understanding of the processing architecture as well as the representation of particular stimuli (Li et al., 2008a; Webster, 2011).

The present paper includes a systematic review about the literature on adaptation effects of face stimuli^1^: which areas does this review on face adaptation cover and which areas does it negotiate? This review focuses on empirical studies that investigate face adaptation effects on a behavioral level; this is typically realized by an overt categorization of face stimuli in a test phase. In fact, we focus on adaptation under optimal conditions in a fully developed and (more or less) optimally functioning cognitive system; i.e., we review findings of studies typically investigating adaptation effects in younger adults possessing face recognition skills that are particularly impressive, for instance the fact that normal persons can discriminate thousands of faces (Jeffery and Rhodes, 2011) when they reach so-called “face expertise” (Schwaninger et al., 2003). This focus on complex objects of the face category is realized in an exclusive and extensive way; that is, we do not relate findings in the area of face adaptation effects to other visual coding mechanisms such as color coding as realized in previous work (Webster, 2011; Webster and MacLeod, 2011). By reviewing face issues exclusively, we assume to provide the main aim of this review most efficiently: we aim to clearly highlight and systematize existing findings as well as gaps in the research literature in the area of face adaptation effects. This systematization should stimulate new directions in face adaptation research and help to design future adaptation studies. In contrast to what we provide, we do not include results about the adaptation of neural processes to face stimuli: for instance, studies on modulations of the N170 as a result of prior adaptation (e.g., Kovács et al., 2006; Kloth et al., 2010) as questions regarding this area of research refer to further dimensions and use different theoretical frameworks, mostly based on specific brain processes and structures. In addition, we omit research from developmental and evolutionary perspectives on face adaptation effects as they were already the major aim of recent alternative review papers (Leopold and Rhodes, 2010; Jeffery and Rhodes, 2011).

We start this review by presenting a framework that enables a systematic organization of findings in the field of face adaptation effects. This framework includes dimensions representing the major characteristics of studies (i.e., experimental manipulations) or operational parameters in this field. As discussed in detail in a later section, the dimensions enable a categorization of the (1) various adapting face information (2) timing characteristics of adaptation effects (e.g., the delay between adaptation and adaptation test phases), and (3) transfer relations between face images presented during these phases (e.g., images of the same or different identities). In the main section of the present review, we discuss the research literature specifically toward each of the framework’s dimensions. Finally, we present options for how to combine findings across different dimensions to demonstrate the relevance of our framework for future studies.

Investigations on adaptation effects in faces are very relevant for the progress of cognitive research, since these effects offer a window into the processes and dynamics of highly complex object processing. First of all, faces are arguably the most important social stimuli since they are the primary means by which we perceive identity information, emotional information, etc. (see below). This expertise and its investigation in adaptation effects provide an essential tool or window for dissecting different levels of neural code and the visual pathway in face processing. This latter fact is also the motivation for a close look at timing and additionally the transfer characteristics of adaptation effects in faces. As research on adaptation effects in faces is moreover a broad and elaborated field today, represented by a great number of adaptation studies employing different procedures and aiming at different research questions, this research field also offers an excellent opportunity for taking a more general perspective on the found effects in the form of a review.

## Framework to Conceptualize Research on Face Adaptation Effects

**Figure 1:**
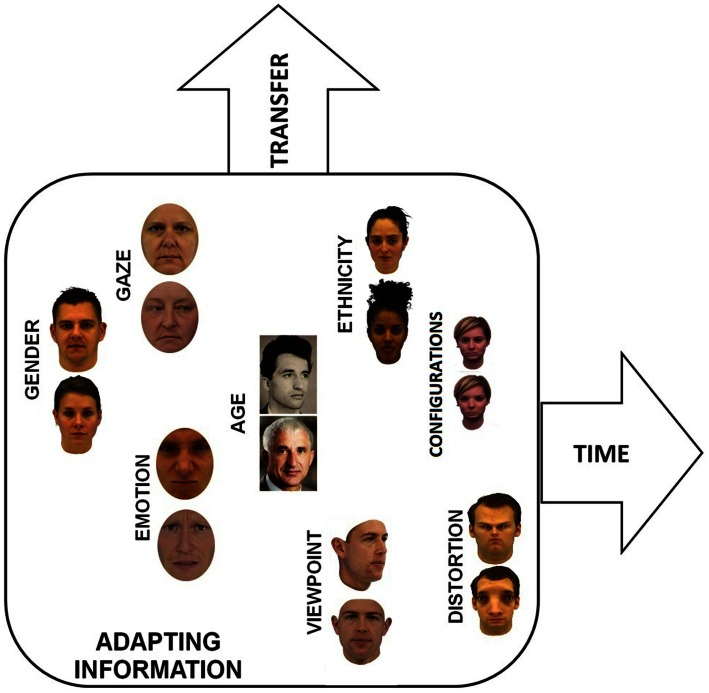
**Framework to review face adaptation effects including dimensions for different types of adapting information, transfer effects, and timing between adaptation and adaptation test phases**.

As illustrated in Figure 1 and in Table 1, we integrate findings in the field of face adaptation research into a conceptual framework that includes three dimensions. The first dimension of this framework represents different types of potential facial information which are susceptible to adaptation; we call this dimension *adapting information*. Instances of adapting information are identity information (e.g., Leopold et al., 2001; Jiang et al., 2006), configural information (e.g., Carbon et al., 2007b; Little et al., 2008), gaze information (e.g., Jenkins et al., 2006), emotional information (e.g., Webster et al., 2004; Ng et al., 2008; Adams et al., 2010), age information (e.g., Schweinberger et al., 2010), gender information (e.g., Kovács et al., 2007; Bestelmeyer et al., 2008), ethnicity information (e.g., Jaquet et al., 2008; Ng et al., 2008), attractiveness information (e.g., Anzures et al., 2009), or viewpoint information (e.g., Chen et al., 2010). The present list of adapting information is completed by face information investigated in the context of face distortion aftereffects (FDAEs; e.g., Webster and MacLin, 1999). This method of manipulating faces may affect types of facial information that are listed above (e.g., configural information, age, identity, gender; please see also later sections). However, FDAEs are realized by unique manipulation algorithms (i.e., distortions by expanding or contracting the frontal-view original face image relative to a midpoint on the nose). Further, these algorithms relax the controlling of which specific information types are affected. For example, manipulating faces in the context of FDAEs affect facial features (e.g., eyes, mouth, etc.) while manipulations of configural information (i.e., spatial distances between these features) rather leave these features unaffected. Generally, these different types of instances of adapting information realize different levels of ecological validity. While differences in age, viewpoint, gaze, or emotion are plausible and realistic in an ecological context, manipulations of identity or configural information have less validity since such changes do not typically occur in the ecosystem.

**Table 1:** **Overview of types of adapting face information and related references**.

Adapting face information	Reference
Age information	O’Neil and Webster (2011), Schweinberger et al. (2010)
Attractiveness information	Anzures et al. (2009), Carbon et al. (2007a), MacLin and Webster (2001), Rhodes et al. (2003), Rhodes et al. (2009b), Webster and MacLin (1999)
Configural information	Carbon and Ditye (2011), Carbon and Leder (2005), Carbon et al. (2007b), Little et al. (2005), Little et al. (2008), McKone et al. (2005), Strobach et al. (2011)
Emotion information	Adams et al. (2010), Benton and Burgess (2008), Fox and Barton (2007), Ng et al. (2008), Webster et al. (2004)
Ethnicity information	Jaquet and Rhodes (2008), Ng et al. (2008), Rhodes et al. (2010), Webster et al. (2004)
Figural (distortion) information	Burkhardt et al. (2010), Hills et al. (2010), Jaquet and Rhodes (2008), Jaquet et al. (2007, 2008), Jeffery et al. (2006, 2007), Morikawa (2005), Robbins et al. (2007), Webster and MacLin (1999), Yamashita et al. (2005), Zhao and Chubb (2001)
Gaze information	Jenkins et al. (2006), Schweinberger et al. (2007)
Gender information	Bestelmeyer et al. (2008), Buckingham et al. (2006), Kovács et al. (2007), Ng et al. (2008), Ng et al. (2006), Webster et al. (2004), Yang et al. (2011)
Identity information	Anderson and Wilson (2005), Hurlbert (2001), Jiang et al. (2006), Leopold et al. (2001), Leopold et al. (2005), Palermo et al. (2011), Rhodes et al. (2009a), Rhodes et al. (2011), Rhodes and Jeffery (2006), Rhodes et al. (2010)
Viewpoint information	Chen et al. (2010), Fang et al. (2007)

The second dimension of the present framework, *time*, orders adaptation effects according to different types of temporal information. The first information type, *delay*, is related to the robustness and sustainability of adaptation effects; basically, the time interval between an adaptation and an adaptation test phase. Delays range from milliseconds (e.g., Leopold et al., 2001; Rhodes et al., 2003) to minutes (e.g., Carbon and Leder, 2006; Kloth and Schweinberger, 2008), but also include days and even weeks under typical laboratory (e.g., Carbon et al., 2007b; Carbon and Ditye, 2011) as well as ecologically more valid test contexts (Carbon and Ditye, 2012). The delay characteristics of adaptation effects are essential for providing useful information about the decay of adaptation effects and thus the “recalibration” and “readaptation” abilities of the visual system (Carbon and Ditye, 2011). Furthermore, they allow inferences about the robustness and consistency of perceptual information in general. In parallel to the “time” information *delay*, we focus on *adaptation duration*, the time span during which the adapting stimulus is presented (e.g., Strobach et al., 2011). Adaptation duration information provides insights into how this time span can modulate the adaptation effect size or the adaptability of faces. Moreover, this type of time information was compared with simple adaptation effects (e.g., with tilt information; Leopold et al., 2005; Rhodes et al., 2007) to explore the dynamics of adaptation effects at different levels of cortical visual hierarchy. Finally, we focus on “time” information of the *test duration* type, establishing the time span during which the test stimulus is presented (e.g., Rhodes et al., 2007). Similar to the adaptation duration, test duration can give insights into to the dynamics of adaptation in face stimuli in contrast to simple adaptation effects.

The third dimension in the present framework is associated with the transfer of adaptation effects. This *transfer* dimension reflects the range and limits of adaptation transfer effects providing important inferences about the nature of processing being linked with specific adapted stimuli or being of more general quality. In this way, the investigation of adaptation is a tool (rather than a topic) for localizing the plasticity and pointing out common coding principles of various levels of visual processing (from retinotopic to high and possibly face-specific levels of visual processing; Webster, 2011; Webster and MacLeod, 2011). There exists two systems of structuring transfer effects: transferring between different (manipulated) image versions of the identical identity during adaptation and test phases (e.g., variations in size or orientation) enables exclusive low-level perceptual effects of adaptation (e.g., on a retinal level; Zhao and Chubb, 2001) to be excluded. Additionally, as proposed by Carbon et al. (2007b), adaptation transfer can be systematically tested with face images used in the adaptation and test phases showing the same images of the same identity vs. different images of the same identity vs. different images of different identities^2^.

The approach can be extended by investigating transfer effects, *inter alia*, across family members, gender, and/or ethnicity.

As will be seen later, not all studies in the field of face adaptation research allow a localization of its research in all dimensions of the applied framework. For example, many studies apply sets of face images of different identities during adaption and the identical set of images during a following test phase (e.g., Buckingham et al., 2006; Chen et al., 2010). Such a procedure prevents conclusions about the transferability of adaptation effects, since potential effects in an adaptation test phase may originate from the presentation of the identical image and/or the presentation of other identities’ images during the adaptation phase.

## Investigating the Adaptation Effects of Different Types of Face Information – The Adapting Information Dimension

Basically, the result patterns in studies on adaptation effects showed an *adaptation bias* and were thus consistent in the following way: values of adaptation test ratings tend toward the (typically extreme) values of adapting information presented during the adaptation phase; in other words, average or neutral faces are perceptually biased away from the adapting face. After introducing findings in the context of FDAEs, we show findings of facial information loosely ordered with increasing abstractedness.

### Face distortion aftereffects

Webster and MacLin (1999) investigated FDAEs within face images of a single identity by presenting adaptation images that were distorted by expanding or contracting the frontal-view original face image relative to a midpoint on the nose. After viewing distorted faces during adaptation (e.g., contracted face images), original faces appear distorted in the direction opposite to the distraction (e.g., expanded face images). In contrast to this effect after adaptations to distorted faces, no such adaptation effect followed the presentation of original face images (i.e., distorted faces still appeared distorted). In this way, Webster and MacLin provided among the first evidence for adaptation effects in complex, natural objects, suggesting that adaptation may play an important normalizing role in face perception and adaptation effects may strongly influence form perception (see also Zhao and Chubb, 2001; Morikawa, 2005; Yamashita et al., 2005; Jeffery et al., 2006, 2007; Jaquet et al., 2007, 2008; Robbins et al., 2007; Jaquet and Rhodes, 2008; Burkhardt et al., 2010; Hills et al., 2010); such a “complex” adaptation phenomenon was recently transferred to animals, trees, or every-day objects (e.g., light bulb; Daelli et al., 2010).

However, the FDAE enables no specification of which types of facial information are precisely involved in face adaptation. For example, the usage of FDAEs simultaneously affects feature information such as mouth, eyes, or eyebrows (e.g., Tanaka and Sengco, 1997; Cabeza and Kato, 2000), configural information such as nose-mouth distance (Young et al., 1987; Rhodes, 1988; Leder and Carbon, 2006), as well as holistic information referring to face processing “as a whole” (e.g., Tanaka and Farah, 1993; Leder and Carbon, 2005). As a consequence, it is essential to systematically vary distinct face information between adaptation and test phases in order to generate precise conclusions about the mechanisms of face adaptation effects, a fact that is not necessarily granted in the context of FDAEs.

### Configural information

Carbon and colleagues (Carbon and Leder, 2005; Carbon et al., 2007b; Carbon and Ditye, 2011; Strobach et al., 2011) aimed instead at investigating adaptation effects of distorted configural information on subsequent adaptation tests. Participants were either presented face images during adaptation of familiar identities with decreased eyes-mouth distance or face images with increased eyes-mouth distance relative to the original. In a subsequent test phase participants were asked to select the veridical version (1) out of a series of versions of gradually altered eyes-mouth distances, or (2) from one original and one slightly altered version (e.g., slightly decreased or increased eyes-mouth distance). The results showed a bias in participants’ selections in the direction of the respective manipulations, e.g., after viewing face images with extremely decreased eyes-mouth distance there was an increased likelihood of selecting a version with slightly decreased distances (for similar results with exclusive shifting the eyes in the vertical axis, see Walton and Hills, 2012). Thus, these studies demonstrated adaptation effects of configural information. Similar results are demonstrated by Little et al. (2005, 2008) following the inspection of manipulated eyes-spacing: inspecting faces with extreme narrow or wide eye distances resulted in increased normality rating for subsequently presented face images with moderately manipulated distances. These latter findings demonstrate the generalization of adaptation effects after exposure to manipulated configural information.

### Gaze and viewpoint information

Adaptation to a consistent leftward or rightward gaze produces ratings that demonstrate an elimination of observers’ perception of the gaze in the adapted direction (Jenkins et al., 2006; Schweinberger et al., 2007). That is, a gaze to the adapted side was subsequently seen as pointing straight. Leftward and rightward viewpoint adaptation resulted in similar adaptation effects (Fang et al., 2007; Chen et al., 2010); thus, a face turned to the adapted side was subsequently seen as pointing straight. Again, these effects can be interpreted as a recalibration mechanism: probably the best heuristic to use if one constantly lacks a straight viewpoint is to retune the processing of gaze direction or viewpoint.

### Emotional and attractiveness information

Another example for testing adaptation effects is represented by investigations on the effects of different facial expressions. For instance, after perceiving a happy face, a previously ambiguous happy-angry face appeared distinctly angry, and thus the boundary between happy and angry faces was shifted toward the happy expression (Webster et al., 2004; Fox and Barton, 2007; Juricevic and Webster, 2012). Such shifted emotion categorization was even evident with adapting stimuli having been suppressed from awareness (Benton and Burgess, 2008; Adams et al., 2010) illustrating the fast and automatic processing of such expressions. Attractiveness adaptation effects demonstrated that viewing consistently distorted faces shifts attractiveness preferences toward the adapting stimuli; for instance, contracted face images (Webster and MacLin, 1999) appeared more attractive after adapting to contracted faces than after adapting to expanded faces (MacLin and Webster, 2001; Rhodes et al., 2003; Carbon et al., 2007a; Anzures et al., 2009). Similarly, faces with left-right asymmetries appeared more attractive when asymmetrical faces were presented during adaptation (Rhodes et al., 2009b).

### Gender information

Adaptation to either masculine or feminine faces increases preferences for novel faces that are (gender-wise) similar to those that were recently seen (Buckingham et al., 2006), as well as increasing the femininity and masculinity ratings of test faces, respectively (see also Webster et al., 2004; Ng et al., 2006, 2008; Kovács et al., 2007). An alternative measurement of gender-adaptation effects demonstrated that adapting to a male/female face selectively enhances discrimination for male/female faces (Yang et al., 2011).

### Age and ethnicity information

When participants viewed young or old adult faces (i.e., adults of different ages), their “young/old boundary” was biased toward the age of the adapting face (O’Neil and Webster, 2011). Consistently, test faces appeared older or younger when the adapting faces were young or old, respectively (Schweinberger et al., 2010). Therefore, there is evidence for an adaptation bias for facial age as well (see also Lai et al., 2012). An analog bias also exists for face ethnicity, exemplarily shown for Caucasian vs. Asian faces: adaptation to an average Asian or Caucasian face reduced identification thresholds for faces from the adapted relative to the unadapted ethnicity (Webster et al., 2004; Rhodes et al., 2010).

### Identity information

**Figure 2:**
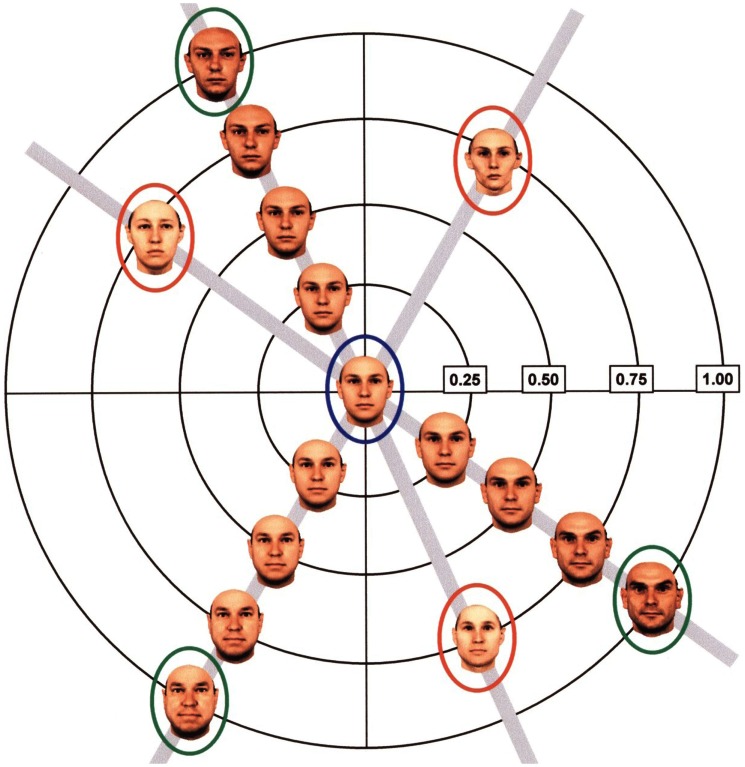
**Computationally derived face space in which the stimuli were generated to investigate face identity adaptation effects (FIAE)**. The original faces (green ellipses) are connected to the average face (blue ellipse) by an “identity trajectory.” Numbers refer to the “identity strength” possessed by the given face (taken from Leopold et al., 2001).

Leopold et al. (2001) provided evidence for increased sensitivity for particular face identities after adaptation, as investigated in the context of face identity aftereffects (FIAEs). Based on the ideas of a “face space” (e.g., a multidimensional representation of faces as their distance to a prototypical “center” face, Valentine, 2001), the authors were able to explain the effect in the theoretical framework of a computationally derived mental representation (Figure 2). After perceiving an “anti-face” (located opposite an original face of an identity, on a trajectory crossing this original face and a face-space average), adaptation specifically shifted perception along a trajectory passing through the adapting anti-face and average face away from the original face, selectively facilitating recognition of a test face lying on this trajectory. Such adaptation effects on the identity level were replicated in a number of studies and variations (Hurlbert, 2001; Anderson and Wilson, 2005; Leopold et al., 2005; Rhodes and Jeffery, 2006; Rhodes et al., 2009a, 2010, 2011; Palermo et al., 2011) and are often explicitly referred to as changes of the face space.

In sum, this section demonstrates adaptation effects of numerous facial attributes. To our knowledge there are no attributes that don’t show any such effects in the research literature, which indicates that adult face coding systems are more flexible than was previously thought (Bruce, 1994). At this point it is essential to stress again, that the class of faces is the only object class that allows investigating and reviewing this large number of information types.

## Investigating Temporal Characteristics of Adaptation Effects – The Time Dimension

### Delay

Face adaptation effects are typically tested with a delay interval of only a few seconds or even less (Webster and MacLin, 1999; Leopold et al., 2001; Rhodes et al., 2003; Benton and Burgess, 2008; Bestelmeyer et al., 2008; Carbon and Ditye, 2011). Given these constraints, the studies mainly show that (A) adapting face information such as face identity occurs on a perceptual level, and (B) there is no recalibration of the visual system after this delay. In sum, they allow conclusions about neither adaptation effects on the representational level nor on the robustness of visual system recalibration. One of the first systematic investigations of adaptation effects and their delay characteristics focused on gaze information (Kloth and Schweinberger, 2008); Table 2 gives an overview of studies testing adaptation effects with short and long delays. This study demonstrated a decrease in the gaze adaptation effect over time but this effect was still measureable up to 385 s after the end of the adaptation phase. This is evidence for the idea that adaptation effects are not completely perceptually-based. Also it suggests that there is no complete “return to normal” (“recalibration,” see Carbon and Ditye, 2011) of the visual system, associated to gaze processing, within this time range of more than 6 min. Consequently, adaptation effects are “stickier” than many of the traditional low-level adaptation effects (e.g., Köhler and Wallach, 1944) and can be initially interpreted as evidence of representation-based effects.

**Table 2:** **Overview of examples of face adaptation studies and their delays between adaptation and test phases**.

Study	Delay
Barrett and O’Toole (2009)	100 ms
Benton and Burgess (2008)	500 ms
Bestelmeyer et al. (2008)	Not available
Carbon and Ditye (2011)	24 h, 1 week
Carbon and Ditye (2012)	1 week
Carbon and Leder (2005)	4,000 ms; 5 min
Carbon and Leder (2006)	80 min
Carbon et al. (2007b)	5 min; 24 h
Fang et al. (2007)	1,000 ms
Hills et al. (2010)	5,000 ms
Hole (2011)	≤2 min
Kloth and Schweinberger (2008)	0–6 min
Kovács et al. (2007)	500 ms
Leopold et al. (2005)	Not available
Leopold et al. (2001)	150; 300; 600; 1,200; 2,400 ms
McKone et al. (2005)	15 min
Rhodes et al. (2007)	1,000 ms
Rhodes et al. (2003)	500 ms
Strobach et al. (2011)	5 min; 24 h
Webster et al. (2004)	250 ms
Webster and MacLin (1999)	Not available

Carbon and colleagues systematically extended research on effects of adaptation to manipulated configural information of famous faces. These studies demonstrated adaptation effects after 5 min (Carbon and Leder, 2005; Carbon et al., 2007b; Strobach et al., 2011), 80 min (Carbon and Leder, 2006), 24 h (Carbon et al., 2007b; Carbon and Ditye, 2011; Strobach et al., 2011), and even 1 week (Carbon and Ditye, 2011). Therefore, adaptation seems to be very robust and refers to effects on the functional level of representations. According to Carbon et al.’s research, it takes at least 1 week for the visual system to return to its original state before adaptation (i.e., to recalibrate to its pre-adaptation state), at least in terms of adaptation effects for configural facial information.

To sum up, regarding the delay dimension, a series of recent experiments revealed relatively long-lasting adaptation effects. This evidence is related to face attributes of gaze information (Kloth and Schweinberger, 2008), and configural information (e.g., Carbon et al., 2007b). It illustrates aspects of face processing that are related to an increased participation of representations and do not only rely on simple iconic traces or simple visual aftereffects (Carbon et al., 2007b). To learn about mental representations, these findings of long-lasting adaptation effects demonstrate that the investigated types of facial information (i.e., gaze information, configural information) are not exclusively processed and coded at a perceptual level. One may speculate that such processing and coding involves long-term memory functions. However, there are no investigations of “delay” effects on facial information aspects of age, attractiveness, emotion, ethnicity, gender, FIAEs, or viewpoint information. Such investigations are essential to assess the functional level (representation level and/or perceptual level), the robustness/sustainability and the time needed for recalibrations of adaptation effects of these types of information. We will come back to this immense gap in the adaptation literature in a later section.

### Adaptation duration

The increase in the time interval for presenting visual adaptation material typically results in an increase of simple perceptual adaptation effects demonstrating the adaptability of tilt, motion, or shape information (Rhodes et al., 2007). In fact, this relation is characterized by a logarithmic function between adaptation duration and effect size. A comparable relation was found in faces. Rhodes, Leopold, Jeffery and colleagues (Leopold et al., 2005; Rhodes et al., 2007) tested the FDAEs as well as FIAEs after varying presentation times of adapting face stimuli; in fact, test stimuli appeared immediately after adaptation material was presented between 1,000 and 16,000 ms. Independent of size relations between adaptation and test faces, FDAEs and FIAEs increased with adaptation time. The relationship between adaptation duration and effect is thus comparable in simple perceptual information as well as complex face objects, illustrating common coding principles at different levels of cortical visual hierarchy.

There were however no, or very short, time delays between adaptation and test phases in these prior studies (Leopold et al., 2005; Rhodes et al., 2007) and thus it is likely that adaptation effects were investigated at the perceptual level only. In contrast, Carbon et al. (2007b) introduced delays of 5 min to 24 h between these phases, allowing investigation of the effects of adaptation delay on the adaptation of memory face representations (in this case, the adapting information was configural information). On the basis of this argument, Strobach et al. (2011) performed multiple regression analyses on the individual adaptation duration and their adaptation effects after both 5 min and 24 h. Positive relations between both measures (i.e., longer presentation times of adaptation faces resulted in increased adaptation effects) demonstrated an impact of the adaptation time on the effect size, extending findings on the perceptual level to findings demonstrating mechanisms instead on a memory level.

There is a lack of studies that explicitly investigate the effects of adaptation time for facial attributes other than FDAEs, FIAEs, and configural information – e.g., for age, gender, and attractiveness. Such investigations would provide elaborated knowledge about the coding principles of face-specific and simple visual information.

### Test duration

Similarly to changes in the magnitude of adaptation effects following variability in adaptation duration, the time span of presenting a test stimulus modulates the magnitude of these effects (Leopold et al., 2005; Rhodes et al., 2007). In fact, test faces were presented for 100, 200, 400, 800, or 1,600 ms and the adaptation effects, as measured in paradigms of FDAEs and FIAEs, reduced with increasing test time. Since similar effects are evident with simple aftereffects, face and simple perceptual information (i.e., tilt, orientation) illustrate common coding principles at different levels of cortical visual hierarchy. However, what is clearly lacking in this domain is research on the question of whether the test duration has an effect when adaptation effects are tested on a memory rather than on a perceptual level. That is to say, it is an open issue in the literature whether the negative relation of test duration and adaptation effect (e.g., increasing test duration and decreasing adaptation effects) is evident after a delay of minutes, hours, or days. Furthermore, this negative relationship was established for FDAEs and FIAEs. However, it is lacking for alternative facial information and such tests should be attempted in future studies. They are essential to establish a more elaborate knowledge of the coding principles of face-specific types of information.

## Investigating the Transferability of Adaptation Effects – The Transfer Dimension

After reviewing adaptation effects of different face information and time characteristics (e.g., the sustainability of adaptation effects), it is essential to review the relationship between the adapting and test face images; i.e., to test transfers of adaptation effects to new face images (or new image versions) not presented during adaptation. Here, we review findings that investigated transfer effects between the same image of one identity (identical images or images differing in viewpoint, orientation, or size between adapting and test images) and different images of the same identity. Additionally, we also discuss adaptation transfer effects between images of different identities. As illustrated in Table 3, we test these transfer effects at the *pictorial level* (identical face image during adaptation and test), *identity level* (different face images of the same identity during adaptation and test), and *novel level* (different face images of different identities during adaptation and test, Carbon and Ditye, 2011). In the following section, we primarily discuss types of adapting face information that demonstrate transfer effects. We followed this strategy because, for the remaining types of face information (e.g., gaze, emotion), there was (A) no investigation of transfer effects and/or (B) no conclusive evidence for such effects.

**Table 3:** **Different transfer levels of adaptation effects as realized in studies of Carbon and colleagues (Carbon et al., 2007b; Carbon and Ditye, 2011, 2012; Strobach et al., 2011)**.

	Transfer of the adaptation effect		
	Picture level	Identity level	Novel level
Adaptation phase	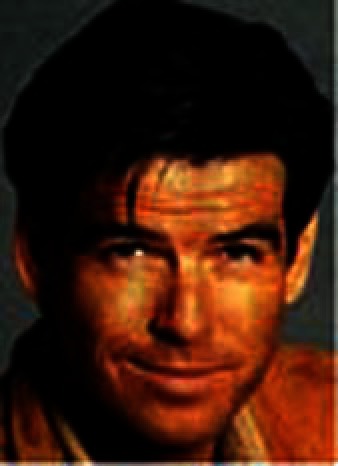	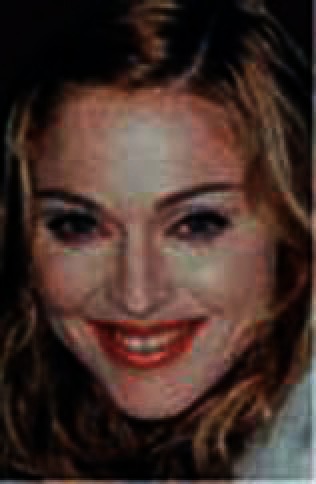	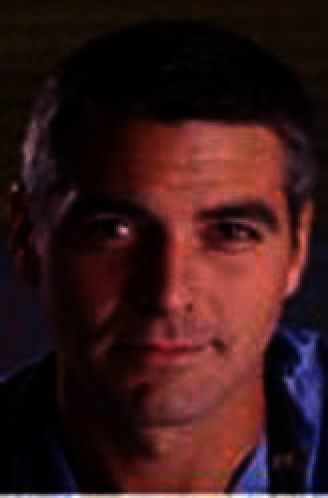
Test phase	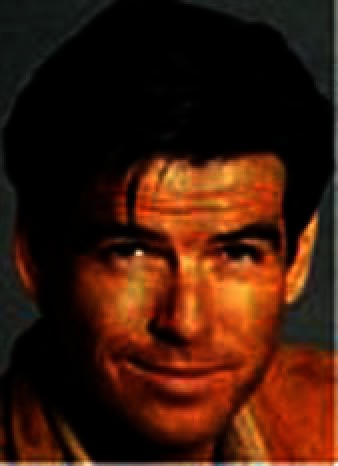	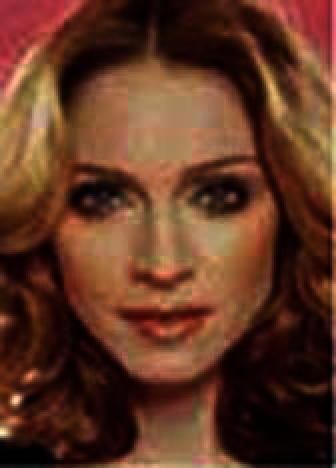	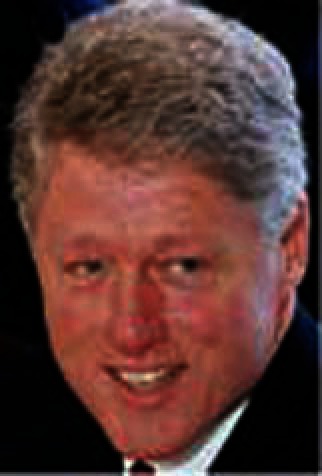

When focusing on FIAEs, Hole (2011) demonstrated adaptation effects with identical face images during adaptation and testing which also transfer onto new image versions changed in viewpoints, orientation, and vertically stretched versions from the adapting face images when using familiar; this confirmed Jiang et al.’s (2006) finding of viewpoint invariance of FIAEs who also added evidence of their transfers across shape and surface reflectance information. Anderson and Wilson (2005) supported Hole’s finding of size independent transfer with unfamiliar, synthetic faces while their study provided no support of a viewpoint-invariant FIAE for this face type. Guo et al. (2009) revealed limits to the transferability of the FIAEs by showing that this effect exclusively works from upright to inverted orientation, but not vice versa with unfamiliar faces. With familiar faces, however, Hole showed that the FIAE produced by inverted adaptation faces and upright test faces was similar to that produced by upright adapting faces. Furthermore, this type of adaptation effect seems to be gender-specific since there is an effect from adaptation to test faces when these faces are related via a gender-specific prototype, whereas there was no such effect with an androgynous face (i.e., combined male and female prototype; Rhodes et al., 2011). Leopold et al. (2001, 2005) showed that relations between a facial prototype and the individual faces in face space could be manipulated by face adaptations of different identities. In other words, this manipulation includes face images during adaptation that are not located on a trajectory crossing an original face and a face-space average (similar to Figure 1); thus, there is evidence for FIAEs on the *novel level*. This conclusion is consistent with the fact that FIAEs are rather high-level perceptual effects: composite faces (different views of a composite face comprised of the top half of a famous face and the bottom half of a non-famous face) either did or did not produce a FIAE depending on whether or not the famous face is explicitly recognized before the post-adaptation test phase (Laurence and Hole, 2012).

Conversely, adaptation to facial expressions (i.e., emotional information) was partly independent from the represented identities. That is, adaptation effects with a focus on facial expressions were transferred to different faces and thus include at least portions of novel-level processing (Fox and Barton, 2007). Interestingly, the above discussed FIAE is not affected by expressional information. That is, FIAEs were not modulated by congruency of facial expression during adaptation and test phases (i.e., same expression vs. different expression; Fox et al., 2008). Thus, expressional adaptation and FIAEs tend toward asymmetry with impact of identity information on expression adaptation, but there is no reverse effect.

For facial gender, Yang et al. (2011) demonstrated that gender discrimination enhancement induced by face adaptation can transfer across a substantial change in three-dimensional facial orientation. Additionally, gender-adaptation effects are position invariant effects (Kovács et al., 2007). These effects also seem to be age-independent, since Barrett and O’Toole (2009) demonstrated an effect of gender adaptation within sets of children’s and adults’ faces and also between these sets of faces. These age-independent effects also demonstrate that gender-adaptation effects may work on a *novel level* since adaptation and test faces were derived from different identities. However, this novel level is limited to the orientation of faces; that is, adaptation effects work independently with upright and inverted face presentations (Watson and Clifford, 2006). Alternatively, the limitation of emotion adaptation effects is set at race boundaries: adapting to one type of emotion in, for instance, a Caucasian face, affects the later processing of an alternative Caucasian face but not that of an alternative ethnicity (i.e., black faces; Otten and Banaji, 2012).

The adaptation effect realized in the form of viewpoint adaptation (i.e., adaptation to left or right-turned faces) occurs at the *novel level* as demonstrated by transfer effects across different identities, different gender, and different vertical orientations (Fang et al., 2007). In the case of face normality ratings and their adaptation effects, there exists evidence for at least orientation-transferable adaptation effects, i.e., between upright and inverted orientations of face images (Rhodes et al., 2003).

Transfer effects of adapting configural information are not only in action at the *pictorial* and *identity* levels, but also at the *novel* level (i.e., different face images of different identities during adaptation and test; Carbon et al., 2007b; Carbon and Ditye, 2011); even though the effect was slightly reduced compared with pictorial and identity conditions. This was demonstrated by the transfer effects of adapting configural information in combinations of identical adaptation and test facial images, of different adaptation and test facial images from the same identity and transfers to new identities, i.e., transfers to test face images of identities not presented during prior adaptation (Walton and Hills, 2012, for comparable results with exclusive eyes shifts in the vertical axis). Little et al. (2008) assumed that such transfer effects are gender-specific. FDAEs are not transferable to images mirrored after the adaptation phase (Morikawa, 2005), but there is evidence of the transfer of such effects to different ethnicities (Jaquet et al., 2007), between different viewpoints (Jeffery et al., 2006, 2007), different orientations of upright and inverted faces (e.g., adapting face is oriented 45° from vertically upright and the test face 45° in the opposite direction; Watson and Clifford, 2003) between different identities and orientations (Webster and MacLin, 1999) as well as different facial image sizes (Zhao and Chubb, 2001; Yamashita et al., 2005). Consequently, FDAEs occur up to the *novel level*. Consistently, adaptation effects of facial age on a *novel level* are demonstrated by transfers between different genders (O’Neil and Webster, 2011) and identities (Schweinberger et al., 2010).

In sum, there is clear evidence for adaptation effects across different identities for a first set of face information (e.g., gender, age, configural information), i.e., transfer effects on a *novel level*. Adaptation of this set of information seems to affect the high-order visual system and/or memory representations. In contrast to these transferable adaptation effects, there is no clear evidence of transfer effects for other face information, such as attractiveness. Furthermore, there is evidence that some face information is only transferable between different identities when the specific subgroups are not changed simultaneously (e.g., FDAE transfers are gender-specific). To learn about processing characteristics and mental representations of faces, this section indicates that face coding is hierarchically structured with an orchestration of common underlying structures. This common structure was demonstrated at the novel level and maybe theoretically represented in a prototype in face space (Valentine, 2001). However, the processing of some facial aspects is characterized by and related to specific modules (e.g., gender-specific modules of FDAEs) potentially working in parallel to a general face-space prototype.

## Future Investigations of Face Adaptation Effects

A summarizing overview of existing and lacking research in the field of face adaptation effects is illustrated in Table 4. Future studies may apply the present framework’s dimensions or operational parameters (adapting information, time, and transfer) for a systematic continuation of investigating face adaptation effects. For instance, for a number of adapting information types (e.g., emotion, age, attractiveness, gender) there exists no test for the robustness of adaptation effects; that is, adaptation effects of these types of face information are demonstrated after short delays between adaptation and adaptation test phases. However, there are no studies that test these adaptation effects after long delays and their decay over time. To present one specific example, in accordance with time intervals applied by Carbon and colleagues (Carbon et al., 2007b; Carbon and Ditye, 2011), the adaptation effect of facial age should be tested after time intervals of 5 min, 24 h, and 1 week in order to cover a broad range of time periods and to test the robustness of the age adaptation effect. Likewise, testing the impact of adaptation and test time should be extended to forms of facial information beyond phenomena investigated in the context of FDAEs (Leopold et al., 2005; Rhodes et al., 2007). As illustrated, a first extension of investigations on adaptation time occurs in the context of adaptation of configural information (Strobach et al., 2011), but other contexts are definitely needed to generate a broader picture of timing aspects in face adaptation. In this way, our framework is able to characterize gaps in the adaptation research literature while combining findings along the dimension adaptation information and delay.

**Table 4:** **Overview of existing and lacking research in the field of face adaptation effects: what types of face information does this research include? What types are neglected?**.

Dimension	Existing investigations on	Lacking investigations on
Adapting information	FDAE	Distinctiveness
	Configural information	Eye color
	Gaze information	
	Viewpoint information	
	Emotional information	
	Attractiveness information	
	Gender information	
	Age information	
	Ethnicity information	
	FIAE	
Time
Delay	Gaze information	Alternative types of information
	Configural information	
Adaptation duration	FDAE	Alternative types of information
	FIAE	Various delays between adaptation and adaptation test phase
Test duration	FDAE	Alternative types of information
	FIAE	Various delays between adaptation and adaptation test phase
Transfer	Configural information	Alternative types of information
	Gender information	Temporal characteristics (i.e., delay, adaptation duration, test duration)
	Emotional information	
	Viewpoint information	
	Attractiveness information	
	FIAE	

Similarly, such an expansion of tests should also be performed on the transfer dimension since this type of test is essential for investigating the functional level of adaptation effects and increasing the ecological validity of these studies. While transfer tests on the same face image of the same identity (e.g., varying orientation or context during adaptation and test; Carbon and Ditye, 2012) enable investigations on picture or “iconic” processing (Carbon, 2008), and thus not necessarily on face processing *per se*, transfer tests on different face images of the same identity as well as different identities’ face images allow for conclusions about the characteristics of face representations. So far, these two types of transfer tests [i.e., (1) different face images of the same identity, (2) different face images of different identities] were separately conducted in most studies. Future studies may combine these two transfer types.

An additional way to continue face adaptation research, in terms of gaining knowledge on the basis of adaptation effects, is to combine aspects (i.e., our framework’s dimensions) of time and transfer. For instance, future studies should systemically investigate the effects of manipulating face information across different delays between adaptation and test phases, combined with tests for the different transfer levels between adaptation and test faces. Additionally, the effects of gender adaptation can be investigated after relatively short and long delays between faces of the same or different age groups, ethnicities, or gaze points. A related investigation focused on transfer effects between different emotional expressions and gender in the context of FDAEs (Tillman and Webster, 2012). This systematic investigation of the interplay of delay and transfer may provide conclusions about the range of adaptation effects and their origin from similar or different methods of neural coding.

Additionally, future studies should relate investigations on the functional level of face adaptation effects to concepts applied to other types of processes or skills. One option might be in relation to improved skills acquired during cognitive training (e.g., working memory training) and their range of transferability. Existing studies on this issue (Li et al., 2008b; Karbach and Kray, 2009; Strobach et al., 2012a,b) categorize the range of skill transfer into near transfer (transfer between situations with common basic characteristics) and far transfer (transfer between situations with structural differences). Conversely, near transfer tests could investigate the adaptation effects of facial images on other facial images, while far transfer tests investigate adaptation effects on facial images after prior adaptation to specific properties of cars (Carbon, 2010) or adaptations to mental sets due to the presentation of gender-typical objects (e.g., lipstick vs. motor bike, Javadi and Wee, 2012). For instance, one could test whether there is a transfer of adaptation effects of configural information (i.e., spatial relations between features) from face stimuli to car stimuli (i.e., far transfer). In particular, front views of cars with a similar setting of parts as can be found in faces are favorable for performing such transfer tests (see Windhager et al., 2010). In fact, this type of future transfer study would cross boundaries between different types of visual objects and may show similarities and differences in these general object characteristics.

## Conflict of Interest Statement

The authors declare that the research was conducted in the absence of any commercial or financial relationships that could be construed as a potential conflict of interest.

## Footnotes

^1^We refer to face adaptation effects as aftereffects (Köhler and Wallach, 1944) at higher perceptual and cognitive levels (Carbon and Ditye, 2011).

^2^In the present work, we define that different facial images can originate from the same identity. For example, the same identity can produce face images differing in emotionality. In contrast, the same facial image of the same identity can produce different versions that vary in size or orientation.
